# Global climate change and infectious diseases.

**DOI:** 10.3201/eid0403.980327

**Published:** 1998

**Authors:** R Colwell, P Epstein, D Gubler, M Hall, P Reiter, J Shukla, W Sprigg, E Takafuji, J Trtanj

**Affiliations:** University of Maryland Biotechnology Institute, College Park, Maryland, USA.


### Global Climate Change and Infectious Diseases

**R. Colwell,* P. Epstein,† D. Gubler,‡ M. Hall,§ P. Reiter,‡ J. Shukla¶, W. Sprigg,# E. Takafuji,** and J. Trtanj§**
*University of Maryland Biotechnology Institute, College Park, Maryland, USA; †Harvard Medical School, Boston, Massachusetts, USA; ‡Centers for Disease Control and Prevention, Atlanta, Georgia, USA; §National Oceanic and Atmospheric Administration, Washington, D.C., USA; ¶Institute of Global Environment and Society, Inc., Calverton, Maryland, USA; #National Research Council, Washington, D.C., USA; **Walter Reed Army Institute of Research, Washington, D.C., USA

Climate change, if it occurs at the level projected by current global circulation models, may have important and far-reaching effects on infectious diseases, especially those transmitted by poikilothermic arthropods such as mosquitoes and ticks. Although most scientists agree that global climate change will influence infectious disease transmission dynamics, the extent of the influence is uncertain. This conference session provided an overview of the issues associated with climate change as it relates to the emergence and spread of infectious diseases.

Two papers set the stage by reviewing data that support or do not support the conclusion that climate change has already influenced transmission of infectious diseases. Some studies support such conclusions as warming at higher elevations, including the retreat of tropical summit glaciers, upward plant displacement, elevational shifts in insect populations and vector-borne diseases, and upward shift of the freezing isotherm (150 m, which is equivalent to 1°C warming) since 1970. Other studies, however, point out that in centuries past, vector-borne diseases such as malaria, dengue, and yellow fever occurred regularly in temperate regions in epidemic form during the summer months. The diseases were eliminated from Europe and North America, and although many areas still have the mosquito vectors, epidemic disease transmission has been prevented by improved living conditions and effective mosquito control. Also, since malaria has historically occurred at elevations of 2,400 m to 2,600 m, its current transmission at high altitudes does not necessarily prove that transmission at these high altitudes is the result of climate change.

The second set of papers provided current evidence of global climate change and described how climatologic data might be used to understand geographic spread and transmission dynamics of an important emerging infectious disease such as cholera. The speakers concluded that global warming is occurring and that weather events appear to be associated with the emergence and spread of cholera in the Americas between 1991 and 1998.

Speakers then focused on the research that will be required to answer the many questions relating to climate change and infectious diseases. They described an effort initiated by the National Oceanic and Atmospheric Administration to take advantage of the strong El Niño Southern Oscillation (ENSO) signal in 1997 to 1998 to study the effect of ENSO on vector-borne diseases. The hypothesis was that ENSO-related changes in precipitation, temperature, and other environmental variables have both direct effects (through drought, flood, and extreme weather events) and indirect effects (through changes in transmission and outbreaks of infectious diseases, particularly diseases transmitted by mosquitoes, rodents, or water) on human health. Diseases studied in the ENSO experiment include cholera in Bangladesh and Peru, cryptosporidiosis in the United States, waterborne and water-related diseases in Florida, marine ecologic disturbances in the eastern United States, dengue in different parts of the world, malaria in Africa, domestic arboviral encephalitides in the United States, and hantavirus pulmonary syndrome in the United States. The National Academy of Sciences and

Institute of Medicine plan to appoint a committee to review critically the published work on this topic and make recommendations for a national research agenda. A number of U.S. government agencies will support this committee financially.

The final presentation addressed the need for cooperation and partnerships in implementing this research agenda. The government agencies involved have unique expertise and perspectives that can be brought to bear on the problem of climate change. Emphasis must be placed on public health intervention measures that are properly implemented and can mitigate the effect of global climate change on infectious disease incidence and geographic spread.

### Suggested Bibliography

1. Epstein PR, Diaz HF, Elias S, Grabherr G, Graham NE, Martens WJM, et al. Biological and physical signs of climate change: focus on mosquito-borne disease. Bulletin of the American Meteorological Society 1998;78:409-17.

Jagadish Shukla
Institute of Global Environment and Society, Inc., Calverton, Maryland, USA

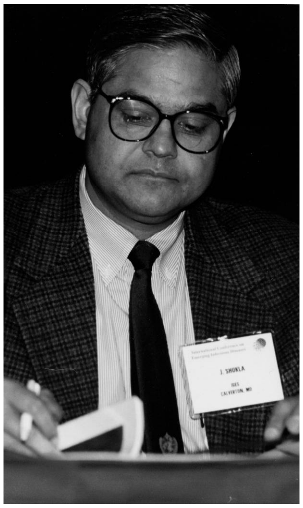

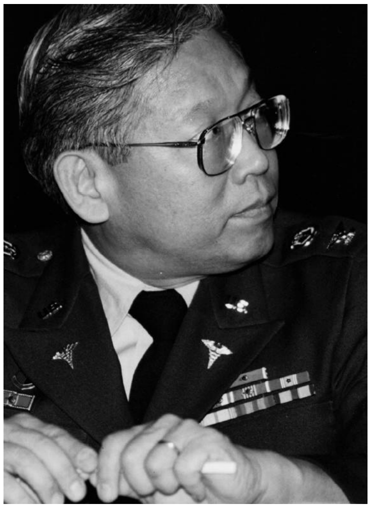

Ernest Takafuji
Walter Reed Army Institute of Research, Washington, D.C, USA

